# Head-to-head comparison of [^18^F]AlF-NOTA-FAPI-04 PET/CT and CE-MRI for diagnosing biliary tract cancer: a retrospective study

**DOI:** 10.3389/fmed.2026.1859832

**Published:** 2026-07-17

**Authors:** Yanyin Zhang, Xiang Peng, Chengzhi Jiang, Jian Yang, Kai Zheng, Hui Ye

**Affiliations:** Department of PET-CT Center, The Affiliated Cancer Hospital of Xiangya School of Medicine, Central South University/Hunan Cancer Hospital, Changsha, China

**Keywords:** [^18^F]AlF-NOTA-FAPI-04 PET/CT, [^18^F]FDG PET/CT, biliary tract cancer, diagnostic performance, MRI

## Abstract

**Purpose:**

To evaluate the diagnostic performance of [^18^F]AlF-NOTA-FAPI-04 PET/CT in detecting primary and metastatic lesions in biliary tract cancer (BTC) and to compare it with contrast-enhanced (CE) MRI.

**Materials and methods:**

A total of 46 patients with suspected BTC were enrolled, all of whom underwent [^18^F]AlF-NOTA-FAPI-04 PET/CT and abdominal CE-MRI examinations. Histopathological findings, typical imaging features, follow-up imaging, and comprehensive clinical assessments were used as reference standards. McNemar’s test was used to compare diagnostic performance; tracer uptake between paired samples was analyzed with the Wilcoxon signed-rank test or paired t-test.

**Results:**

In total, 46 participants (mean age 61.5 ± 9.91 years) were evaluated. In the patient-based comparison, [^18^F]AlF-NOTA-FAPI-04 PET/CT and CE-MRI demonstrated comparable sensitivity in detecting primary tumors (42/44 [95%] vs. 44/44 [100%], *p* = 0.480), lymph node metastases (20/21 [95%] vs. 18/21 [86%], *p* = 0.480), and distant metastases (12/12 [100%] vs. 9/12 [75%], *p* = 0.064). However, in detecting lymph node metastases, [^18^F]AlF-NOTA-FAPI-04 PET/CT achieved higher specificity (25/25 [100%] vs. 19/25 [76%], *p* = 0.041) and accuracy (45/46 [98%] vs. 37/46 [80%], *p* = 0.013) than CE-MRI.

**Conclusion:**

The findings of this study demonstrate that [^18^F]AlF-NOTA-FAPI-04 PET/CT and abdominal CE-MRI exhibit comparable sensitivity in detecting primary lesions and metastases in BTC. However, [^18^F]AlF-NOTA-FAPI-04 PET/CT demonstrated superior specificity and accuracy in lymph node detection and effectively overcame the limitations of abdominal CE-MRI in evaluating distant metastases across the whole body.

## 1 Introduction

Biliary tract cancers (BTC), including intrahepatic, perihilar, and distal cholangiocarcinoma, as well as gallbladder cancer, constitute a biologically aggressive group of malignancies with rising incidence and persistently poor prognosis (1). Surgical resection offers the best chance for long-term survival in BTC, requiring reliable imaging for diagnosis and accurate staging.

Computed tomography (CT) and magnetic resonance imaging (MRI) are the principal imaging modalities for the diagnosis of BTC (2). CT provides high spatial resolution cross-sectional images that facilitate assessment of tumor characteristics and distant metastases. MRI, owing to its superior soft-tissue contrast, offers more detailed delineation of the biliary anatomy and the local extent of tumor infiltration (3). However, CT and MRI are both morphological techniques, and they cannot directly describe the molecular characteristics of tumors.

Fluorine 18 [^18^F] fluorodeoxyglucose positron emission tomography/computed tomography ([^18^F]FDG PET/CT) plays a pivotal role as a functional imaging modality, with significant implications in staging, restaging, treatment planning, and therapy response evaluation for various cancers (4). However, its diagnostic performance in BTC remains suboptimal, with extrahepatic cholangiocarcinoma being particularly susceptible to false-negative results. This limited sensitivity is primarily attributable to low tumor cellularity, the characteristic infiltrative thin-layer growth pattern along the biliary tract, and specific histologic variants, including mucinous adenocarcinoma, that intrinsically show reduced FDG avidity (5). Furthermore, the limited accuracy of [^18^F]FDG PET/CT in detecting nodal, hepatic, and peritoneal metastases in BTC reduces its utility in clinical decision-making (6, 7).

Fibroblast activation protein (FAP), a type II transmembrane serine protease, is specifically overexpressed in various epithelial tumors, including BTC, while exhibiting little to no expression in healthy tissues (8, 9). This differential expression makes FAP inhibitor (FAPI) PET/CT a promising modality for BTC, and multiple studies show that [^68^Ga]FAPI PET/CT achieves higher lesion detection rates and superior semi-quantitative metrics than [^18^F]FDG PET/CT (7, 10). However, the widespread adoption of [^68^Ga]FAPI PET/CT is constrained by several limitations, including its short half-life (68 min), suboptimal spatial resolution, and limited availability of ^68^Ge/^68^Ga generators. In contrast, the [^18^F]-labeled tracer [^18^F]AlF-NOTA-FAPI-04 can be produced in large quantities via cyclotron, facilitating centralized distribution over long distances. It has already shown excellent tumor imaging performance in multiple clinical evaluations (11, 12).

Despite reports on the use of [^68^Ga]FAPI PET/CT in cholangiocarcinoma, data on the [^18^F]-labeled analogue [^18^F]AlF-NOTA-FAPI-04 in BTC remain scarce. In this study, we systematically compare the diagnostic performance of [^18^F]AlF-NOTA-FAPI-04 PET/CT versus abdominal CE-MRI for detecting primary tumors, lymph nodes, and distant metastases in BTC.

## 2 Materials and methods

### 2.1 Patients

This is a retrospective study that has been reviewed and approved by the institutional review board of the Hunan Cancer Hospital and registered at ClinicalTrials.gov (NCT06557590). Between March 2023 and August 2025, patients with suspected BTC were screened for eligibility. Inclusion criteria were as follows: (1) adult patients (aged ≥ 18 years); (2) patients who underwent both [^18^F]AlF-NOTA-FAPI-04 PET/CT and abdominal CE-MRI for initial staging within 2 weeks, and did not receive any treatment between the two exams. Exclusion criteria were as follows: (1) patients with more than two primary tumors; (2) patients without complete clinical or pathological records; (3) patients who cannot cooperate to complete both examinations. Ultimately, 46 patients who met the criteria were enrolled, and all provided written informed consent. Among these, 5 patients additionally underwent [^18^F]FDG PET/CT at our institution during the same study period, enabling a comparative analysis of the two tracers.

### 2.2 Radiopharmaceuticals

[^18^F] was generated on site via proton irradiation (9.8 MeV) of O-18-H2O using a MINItrace cyclotron (GE HealthCare, Milwaukee, WI, USA). The FAPI-04 precursor was obtained from PET Science and Technology CO., LTD (Beijing, China). Radiolabeling of NOTA-FAPI-04 with ^18^F through AlF complexation was conducted according to the protocol described by Jiang et al. (13). [^18^F]FDG was synthesized using the standard procedure with a dedicated [^18^F]FDG synthesis module (AIO, TRSIS, China). Both [^18^F]AlF-NOTA-FAPI-04 and [^18^F]FDG achieved radiochemical purity greater than 95%. All preparations were sterile and pyrogen-free and met the release criteria for human administration.

### 2.3 [^18^F]AlF-NOTA-FAPI-04 PET/CT and [^18^F]FDG PET/CT acquisition

All patients underwent [^18^F]AlF-NOTA-FAPI-04 PET/CT, and five of them additionally underwent paired [^18^F] FDG PET/CT within 1 week. PET/CT was acquired 40–60 min after intravenous injection of [^18^F] AlF-NOTA-FAPI-04 (3.7 MBq [0.1 mCi]/kg). For [^18^F]FDG PET/CT, participants fasted for at least 6 h, and their blood glucose levels were measured and confirmed to be < 11.1 mmol/L before intravenous administration of the radiopharmaceutical. PET/CT was performed approximately 60 min after [^18^F]FDG injection (3.7 MBq [0.1 mCi]/kg). For both procedures, patients drank about 500 mL of water before scanning. All acquisitions were performed on a PET/CT scanner (Discovery MI, GE HealthCare, Milwaukee, WI, USA). For [^18^F]AlF-NOTA-FAPI-04 and [^18^F]FDG PET/CT, the imaging field of view extended from the vertex to mid-thigh. A low-dose CT scan was performed using 64-slice helical CT with the following settings: 110 kV; 200-360mAs with automated dose modulation; slice: 3.75 mm; matrix: 256 × 256; 0.5-s rotation time; pitch index 0.984. All acquired data were transferred to the Advantage Workstation (AW 4.7, GE HealthCare), and were reconstructed using a Bayesian penalized likelihood reconstruction method (Q. Clear, GE HealthCare) with *β* = 750.

### 2.4 MRI acquisition

All patients underwent upper abdominal MRI examinations using a 3.0 T MRI scanner (Achieva; Philips Healthcare, Netherlands). The scanning range extended from the diaphragmatic dome to the lower pole of the kidneys. Initial non-contrast axial imaging was performed, including T1-weighted imaging (repetition time [TR] = 3.00 ms, echo time [TE] = 1.40 ms, field of view [FOV] = 338 mm, matrix = 246 × 248, slice thickness = 3 mm), T2-weighted imaging (TR = 1297.92 ms, TE = 70.00 ms, FOV = 361 mm, matrix = 218 × 220, slice thickness = 5 mm) and diffusion-weighted imaging (DWI, b = 0 and 800 s/mm^2^). Apparent diffusion coefficient (ADC) maps were automatically generated by the scanner software. Of note, DWI/ADC sequences were available in 41 of the 46 enrolled patients (89.1%); in the remaining 5 patients, these sequences were not acquired owing to clinical scheduling constraints. Subsequently, contrast-enhanced axial and coronal T1-weighted imaging was conducted following intravenous administration of gadodiamide (Gd-DTPA-BMA) at a dose of 0.2 mL/kg body weight, using identical sequence parameters to the non-contrast scans.

### 2.5 Image evaluation

Semi-quantitative analysis of all [^18^F]AlF-NOTA-FAPI-04 PET/CT and [^18^F]FDG PET/CT scan data was performed using the Advantage workstation. For all primary and metastatic lesions, regions of interest (ROIs) were delineated on axial slices to encompass regions of increased uptake and were automatically integrated into three-dimensional volumes of interest (VOIs) for quantitative analysis. The maximum standardized uptake value (SUVmax) was automatically calculated for each lesion. Additionally, VOIs were manually placed on the mediastinal blood pool (descending aorta) and on non-involved regions of the right hepatic lobe to automatically derive the mean standardized uptake value (SUVmean) of the background. The tumor-to-background ratio (TBR) was calculated using the SUVmax of each lesion relative to the SUVmean of the mediastinal blood pool (TBR_blood_) and the SUVmean of the liver (TBR_liver_).

In the analysis of PET/CT images, a positive lesion was defined as a focal area of tracer uptake significantly higher than the adjacent normal background activity, while excluding known physiological uptake and confirmed benign lesions. Conversely, if the tracer uptake in the lesion area did not exceed the background tissue, it was classified as negative. The MRI evaluation of BTC and its metastatic lesions was conducted in accordance with the criteria reported in the previous literature (14, 15). All images were independently reviewed by two experienced nuclear medicine physicians, each with over 10 years of expertise in oncology. In cases of diagnostic discrepancies, consensus was achieved through discussion. For all lesions subjected to biopsy or surgical resection, the final diagnosis was confirmed using pathological results as the gold standard. For tumor lesions that were not biopsied, the diagnosis was verified through a combination of clinical follow-up (≥ 6 months) and progression characteristics observed in conventional imaging modalities, including ultrasound, CT, or MRI (16).

### 2.6 Statistical analysis

Statistical analyses were conducted using IBM SPSS Statistics for Windows, version 27 (IBM Corp., Armonk, NY, USA). Statistical significance was defined as a two-tailed *p*-value < 0.05. Categorical variables were reported as frequencies and percentages, and continuous variables were presented as mean ± standard deviation (SD) or median with interquartile range (Q25-Q75), according to distributional characteristics. Comparisons of sensitivity, specificity, and accuracy were conducted using the McNemar test. For two-group data conforming to a normal distribution, the paired t-test was applied; for non-normally distributed two-group data, the Wilcoxon signed-rank test was utilized. Additionally, comparisons among three or more independent groups were performed using the Kruskal-Wallis test.

## 3 Results

### 3.1 Patient characteristics

A total of 72 patients were evaluated during the study period. Of these, 26 were excluded for lacking pathological confirmation, resulting in 46 newly diagnosed patients being enrolled. All enrolled patients successfully underwent [^18^F]AlF-NOTA-FAPI-04 PET/CT and abdominal CE-MRI, with no serious imaging-related adverse events reported. Baseline clinical characteristics are summarized in Table 1. The cohort comprised 25 males and 21 females, with a mean age of 61.5 ± 9.91 years. A total of 21 patients underwent surgical treatment, while 25 patients underwent biopsy of the primary tumor for histopathological diagnosis (Figure 1).

**Table 1:** Patient characteristics.

Characteristic	Value
No. of participants	46
Age (y)*	61.5 ± 9.91 (36–77)
Sex	
F	21 (46%)
M	25 (54%)
Location	
Intrahepatic Bile Duct	18 (39%)
Perihilar Bile Duct	10 (22%)
Distal Bile Duct	16 (35%)
Gallbladder	2 (4%)
Post-scan treatment	
Surgery	21 (46%)
Chemotherapy	13 (28%)
None	12 (26%)
Pathologic types	
Well and moderately differentiated adenocarcinoma	12 (26%)
Poorly differentiated adenocarcinoma	18 (39%)
Adenocarcinoma, not otherwise specified (NOS)	12 (26%)
Moderately differentiated squamous cell carcinoma	1 (2%)
EBV-associated	1 (2%)
Cholangitis	2 (4%)
Reference standards	
Surgical resection	21 (46%)
Pathological biopsies	25 (54%)

*Age is expressed as the mean ± SD, with range in parentheses. Note. Percentages may not sum to 100% due to rounding.

**Figure 1:**
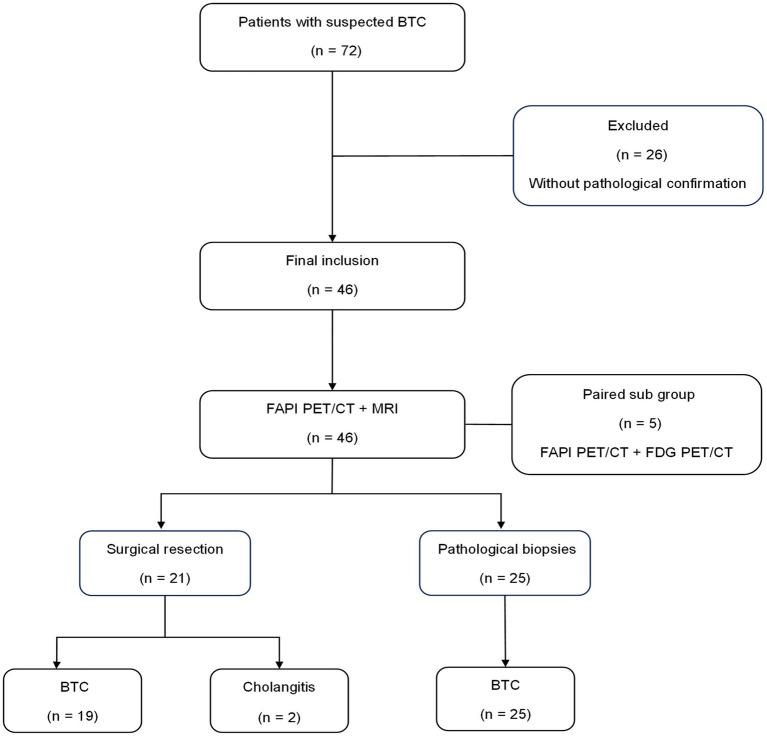
Study flowchart.

### 3.2 Tumoral uptake on [^18^F]AlF-NOTA-FAPI-04 PET/CT

The mean longest diameter of the primary tumor was 4.5 ± 2.0 cm (range: 1.1–8.6 cm), with SUVmax and TBR values summarized in Table 2. The results showed that the median SUVmax (IQR) of the primary tumor was 14.3 (11.2, 19.1), indicating high radiotracer uptake. In BTC, the uptake on [^18^F]AlF-NOTA-FAPI-04 PET/CT did not demonstrate statistically significant differences among various anatomical locations (*p* = 0.695) or histological differentiation grades (*p* = 0.599). Consequently, no additional pairwise comparisons were performed. However, the TBR_blood_ of distal cholangiocarcinoma was significantly higher than the TBR_liver_ (*p* = 0.013). For metastatic lesions, including lymph nodes and distant metastases, no significant differences were found in TBR values (*p* > 0.05).

**Table 2:** Semiquantitative parameters [^18^F]AlF-NOTA-FAPI-04 PET/CT in BTC.

Tumor Lesions and Parameters	No. of lesions	SUV_max_	TBR_blood_	TBR_liver_	*p*-value^†^
Primary tumors	42	14.3 (11.2, 19.1)	11.5 (8.8, 15.7)	8.8 (3.8, 13.1)	0.081
ICC	18	13.0 (10.8, 18.0)	10.9 (7.9, 14.4)	11.0 (8.0, 14.8)	0.586
PCC	10	15.3 (12.1, 20.6)	12.5 (10.4, 16.2)	8.1 (3.2, 14.2)	0.059
DCC	12	13.8 (10.7, 19.5)	12.7 (7.2, 16.0)	5.0 (3.2, 9.3)	0.013
GBC^*^	2	12.4, 18.0	6.2, 11.3	3.2, 20.0	0.655
*p*-value^‡^		0.695	0.496	0.183	
Lymph node metastases	N/A	12.4 (9.1, 15.0)	10.7 (6.5, 13.8)	7.0 (3.2, 14.6)	0.231
Distant metastases	N/A	9.5 (7.8, 15.9)	6.9 (5.7, 10.7)	7.3 (3.2, 14.2)	0.859

ICC, intrahepatic cholangiocarcinoma; PCC, perihilar cholangiocarcinoma; DCC, distal cholangiocarcinoma; GBC, gallbladder carcinoma.

^†^*p*-value for comparing TBR_blood_ and TBR_liver_ using the Wilcoxon signed-rank test.

^‡^*p*-value for comparing SUVmax and TBR across the four anatomical BTC subgroups using the Kruskal–Wallis test.

^*^Data presented as individual values due to the small sample size in the GBC group.

N/A not applicable, There was extensive and diffuse lymphadenopathy as well as distant metastases, making it impossible to count the lesions individually.

### 3.3 Diagnostic performance of [^18^F]AlF-NOTA-FAPI-04 PET/CT and CE-MRI for primary tumors in BTC

The diagnostic performance of [^18^F]AlF-NOTA-FAPI-04 PET/CT was evaluated in comparison with CE-MRI, as summarized in Table 3. In the patient-based analysis, the sensitivity and accuracy of [^18^F]AlF-NOTA-FAPI-04 PET/CT and CE-MRI for primary lesion detection were 95% vs. 100% (*p* = 0.480) and 91% vs. 98% (*p* = 0.248), respectively (Figures 2–4). [^18^F]AlF-NOTA-FAPI-04 PET/CT identified two false-positive cases in the common bile duct region, which were confirmed as cholangitis by postoperative pathology (Figure 5). Additionally, two false-negative cases were also observed (Figure 6). In one case, physiological biliary excretion of the tracer resulted in intense radioactivity in the biliary tree, which obscured the target lesion. In the other case, biliary obstruction led to secondary pancreatitis, accompanied by markedly increased inflammatory FAPI uptake in the pancreatic parenchyma, which masked the primary tumor.

**Table 3:** Diagnostic performance of [^18^F]AlF-NOTA-FAPI-04 PET/CT and CE-MRI on a participant basis.

	Sensitivity (%)	Specificity (%)	Accuracy (%)	PPV (%)	NPV (%)
Primary tumors					
FAPI-PET/CT	95 (42/44)	N/A (0/2)	91 (42/46)	95	N/A
CE-MRI	100 (44/44)	50 (1/2)	98 (45/46)	98	100
*p*-value	0.480	N/A	0.248	0.250	N/A
Lymph node metastases					
FAPI-PET/CT	95 (20/21)	100 (25/25)	98 (45/46)	100	96
CE-MRI	86 (18/21)	76 (19/25)	80 (37/46)	75	86
*p*-value	0.480	0.041	0.013	0.008	0.008
Distant metastases					
FAPI-PET/CT	100 (12/12)	88 (30/34)	91 (42/46)	75	100
CE-MRI	75 (9/12)	94 (32/34)	89 (41/46)	82	91
*p*-value	0.064	0.391	0.722	0.625	0.234

Comparisons for primary tumors and lymph nodes were limited to the abdominal region (CE-MRI field of view); for distant metastases, whole-body PET/CT was compared with abdominal CE-MRI. N/A, not applicable, Due to the very small number of benign controls, the estimated specificity is unstable and not considered a valid performance metric.

**Figure 2:**
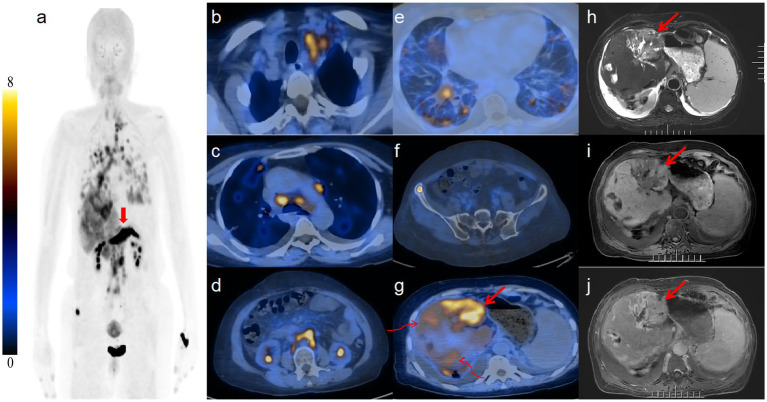
A 53-year-old female presented with a two-month history of abdominal pain. **(a)** The maximum intensity projection (MIP) image from [^18^F]AlF-NOTA-FAPI-04 PET/CT demonstrates widespread FAPI-04-avid nodules. **(b–g)** These include a primary lesion located in the lateral segment of the left hepatic lobe (Straight Arrow, SUVmax = 11.5), multiple enlarged lymph nodes in the left supraclavicular region, chest, and abdomen, bilateral pulmonary lymphangitic carcinomatosis and a right iliac bone metastasis. **(h–j)** The primary lesion is also clearly visualized on MRI (Straight Arrow), with biopsy confirming a diagnosis of intrahepatic cholangiocarcinoma. **(a,g)** Additionally, [^18^F]AlF-NOTA-FAPI-04 PET/CT revealed intense pancreatic uptake associated with inflammation secondary to biliary obstruction (thick arrow, SUVmax = 17.1) and heterogeneously increased hepatic uptake due to cirrhosis (curved arrow, SUVmax = 8.3).

**Figure 3:**
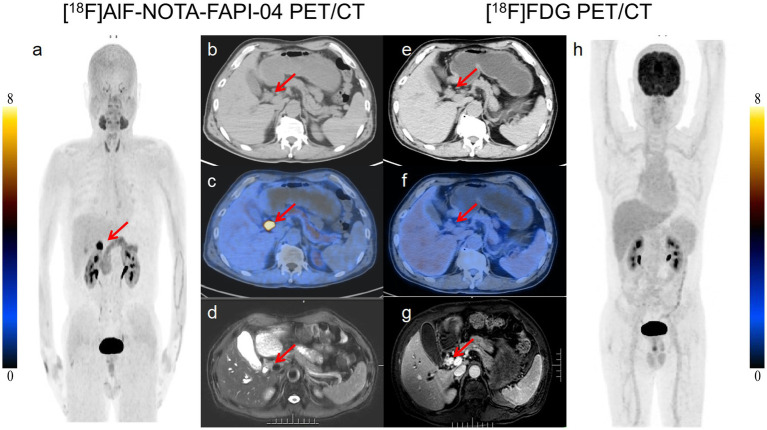
A 58-year-old male presented with a one-month history of abdominal distension accompanied by jaundice. **(a–c)** [^18^F]AlF-NOTA-FAPI-04 PET/CT revealed intense tracer uptake in the region of the common hepatic duct (SUVmax = 21.0). **(e,f,h)** The corresponding area exhibited minimal uptake on [^18^F]FDG PET/CT (SUVmax = 2.2). **(d,g)** MRI showed a slightly hyperintense signal on axial T2-weighted imaging, accompanied by marked enhancement on contrast imaging. Postoperative pathology confirmed the diagnosis of well-differentiated cholangiocarcinoma.

**Figure 4:**
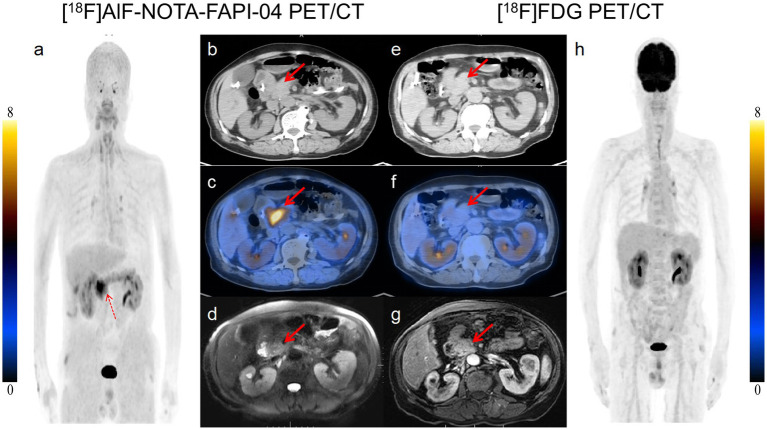
A 68-year-old male presented with hematochezia and underwent further investigation. **(a–c)** [^18^F]AlF-NOTA-FAPI-04 PET/CT demonstrated intense tracer uptake at the distal common bile duct (SUVmax = 10.8). **(e,f,h)** In contrast, the corresponding site showed minimal uptake on [^18^F]FDG PET/CT (SUVmax = 2.4). **(d,g)** MRI showed a slightly hyperintense signal on axial T2-weighted imaging, with moderate enhancement observed on contrast-enhanced scans. Postoperative pathological examination confirmed the diagnosis of distal cholangiocarcinoma.

**Figure 5:**
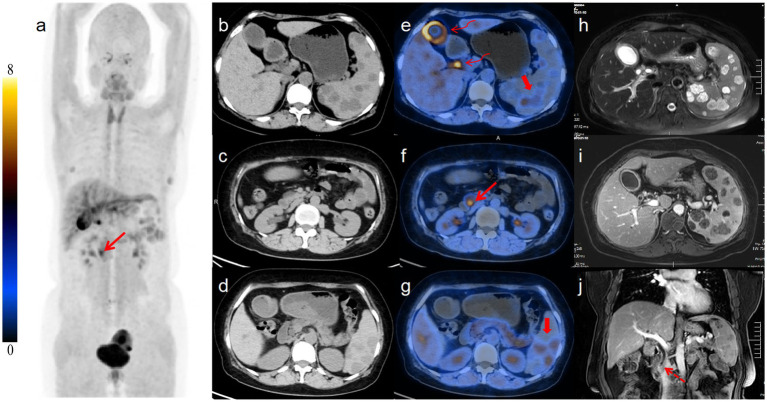
A 53-year-old female presented with abdominal distension, pain, and jaundice for further evaluation. **(a,c,f)** [^18^F]AlF-NOTA-FAPI-04 PET/CT demonstrated intense tracer uptake at the distal end of the common bile duct (straight arrow, SUVmax = 10.8). **(e)** High tracer uptake was also observed in the gallbladder wall and cystic duct (curved arrow, SUVmax = 13.4). **(b,d,e,g)** Multiple hypodense nodules in the spleen exhibited variably increased uptake (thick arrow, SUVmax = 5.1). **(h–i)** Corresponding MRI revealed multiple nodules with abnormal signal intensity, showing mild enhancement on contrast imaging, consistent with hemangiomas. **(j)** Coronal contrast-enhanced imaging indicated slight wall thickening and enhancement of the distal common bile duct (dashed arrow). Postoperative histopathology confirmed cholelithiasis with cholecystitis and choledocholithiasis with cholangitis.

**Figure 6:**
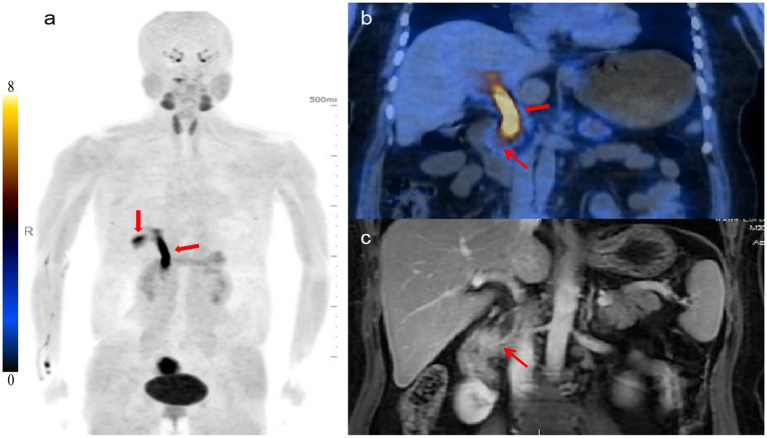
A 47-year-old female with a biopsy-confirmed diagnosis of well-differentiated cholangiocarcinoma underwent [^18^F]AlF-NOTA-FAPI-04 PET/CT for staging assessment. **(a)** The MIP image of [^18^F]AlF-NOTA-FAPI-04 PET/CT demonstrates physiological high uptake of bile in the gallbladder and common bile duct (thick arrow). **(b)** [^18^F]AlF-NOTA-FAPI-04 PET/CT shows no significant tracer uptake at the distal segment of the common bile duct (straight arrow), while physiological uptake of bile is observed in the dilated proximal common bile duct (thick arrow). **(c)** Coronal contrast-enhanced MRI reveals wall thickening and enhancement at the distal common bile duct (straight arrow).

### 3.4 Diagnostic performance of [^18^F]AlF-NOTA-FAPI-04 PET/CT and CE-MRI in lymph node metastases

For the evaluation of abdominal regional lymph node metastases, [^18^F]AlF-NOTA-FAPI-04 PET/CT significantly outperformed CE-MRI in specificity (100% vs. 76%, *p* = 0.041), accuracy (98% vs. 80%, *p* = 0.013), PPV (100% vs. 75%, *p* = 0.008), and NPV (96% vs. 86%, *p* = 0.008), while demonstrating comparable sensitivity (95% vs. 86%, *p* = 0.480). Notably, all 21 cases of lymph node metastases involved abdominal lymph nodes. Consequently, the counts for true positive (TP), true negative (TN), false positive (FP), and false negative (FN), along with the corresponding diagnostic performance metrics, remained identical whether the analysis was restricted to abdominal PET/CT or extended to whole-body PET/CT. Additionally, [^18^F]AlF-NOTA-FAPI-04 PET/CT further identified distant lymph node metastases beyond the abdominal region in 8 patients, involving the cervical, thoracic, retroperitoneal, and pelvic regions. Lymph node metastases were confirmed through surgical pathology (*n* = 3), biopsy pathology (*n* = 1), or imaging follow-up (*n* = 17). Meanwhile, CE-MRI yielded 6 false-positive results for lymph node metastases. Additionally, one case of lymph node metastases was confirmed by postoperative pathology but was missed by both [^18^F]AlF-NOTA-FAPI-04 PET/CT and CE-MRI, representing a false-negative finding.

### 3.5 Diagnostic performance of [^18^F]AlF-NOTA-FAPI-04 PET/CT and CE-MRI distant metastases

Regarding the diagnostic diagnostic performance for distant metastases, given that the contingency table counts (TP/TN/FP/FN) in the paired analysis confined to the abdominal region were identical between the two imaging modalities, we expanded the comparison to include whole-body [^18^F]AlF-NOTA-FAPI-04 PET/CT versus abdominal CE-MRI to better align with clinical staging practices. The sensitivity, specificity, and accuracy of [^18^F]AlF-NOTA-FAPI-04 PET/CT were 100, 88, and 91%, respectively, compared to 75, 94, and 89% for CE-MRI. No statistically significant differences were observed between the two modalities (*p* = 0.064, *p* = 0.391, and *p* = 0.722, respectively). A total of 12 cases were identified with distant metastases, including one case confirmed by biopsy pathology and 11 cases verified through imaging follow-up. Among these, [^18^F]AlF-NOTA-FAPI-04 PET/CT and CE-MRI demonstrated comparable detection capabilities in 6 cases (3 cases of liver metastases and 3 cases of peritoneal metastases). In the remaining 6 cases, [^18^F]AlF-NOTA-FAPI-04 PET/CT outperformed CE-MRI by detecting additional distant metastases outside the abdominal region, with 3 cases exclusively identified by [^18^F]AlF-NOTA-FAPI-04 PET/CT. The metastatic sites outside the abdominal region included the lungs (*n* = 4), pleura (*n* = 2), bones (*n* = 3), and ovaries (*n* = 1). In addition, [^18^F]AlF-NOTA-FAPI-04 PET/CT yielded four false-positive findings: one left scapular lesion and one meningeal nodule (both non-progressive over 6 months), one left rib lesion subsequently confirmed to represent fracture healing, and multiple splenic lesions interpreted as hemangiomas on CE-MRI; postoperative pathology of the primary tumor indicated cholangitis-related changes (Figure 5). CE-MRI yielded two false-positive findings: one hepatic lesion and one left rib lesion.

### 3.6 Comparison of [^18^F]AlF-NOTA-FAPI-04 PET/CT and [^18^F]FDG PET/CT uptake values

In a paired subgroup of patients who underwent both [^18^F]FDG and [^18^F]AlF-NOTA-FAPI-04 PET/CT imaging (*n* = 5), we compared the SUVmax of the lesions and their TBR (Table 4). In all three cases of distal cholangiocarcinoma (dCC), [^18^F]AlF-NOTA-FAPI-04 PET/CT demonstrated markedly higher SUVmax, TBRblood, and TBRliver values compared with [^18^F]FDG PET/CT (Figures 3, 4). Similarly, in the two cases of cholangitis, [^18^F]AlF-NOTA-FAPI-04 PET/CT also showed pronounced uptake (Figure 5), with SUVmax, TBRblood, and TBRliver values exceeding those observed on [^18^F]FDG PET/CT. However, due to the limited sample size, inferential statistical analysis was not performed.

**Table 4:** Comparison of tracer uptake between [^18^F]AlF-NOTA-FAPI-04 and [^18^F]FDG on PET/CT (*n* = 5).

Patient no.	Age (y)	Sex	Histology	SUV_max_		TBR_blood_		TBR_liver_	
				FAPI	FDG	FAPI	FDG	FAPI	FDG
19	62	M	DCC	14.0	4.8	11.7	2.3	4.4	1.6
31	68	M	DCC	10.8	2.4	9.0	1.3	5.1	1.3
41	58	M	DCC	21.0	2.2	12.4	0.9	10.0	0.8
42	75	M	Cholangitis	8.3	2.1	4.2	1.1	1.5	0.9
43	53	F	Cholangitis	10.8	2.2	8.3	0.8	5.1	0.7

DCC, distal cholangiocarcinoma.

## 4 Discussion

BTC is aggressive malignancy with poor prognosis, making early detection and accurate staging crucial for effective management. The patient-based comparative analysis in this study demonstrated that the two imaging modalities exhibited comparable sensitivity (*p* = 0.480) and accuracy (*p* = 0.248) in detecting primary tumors. In the evaluation of lymph nodes, [^18^F]AlF-NOTA-FAPI-04 PET/CT showed a sensitivity similar to that of MRI, but significantly outperformed MRI in terms of specificity (*p* = 0.041) and accuracy (*p* = 0.013). Moreover, [^18^F]AlF-NOTA-FAPI-04 PET/CT detected additional distant lymph node metastases in the cervical, thoracic, retroperitoneal, and pelvic regions. Although no statistically significant differences were observed between the two modalities in detecting distant metastases (all *p* > 0.05), [^18^F]AlF-NOTA-FAPI-04 PET/CT successfully identified three additional cases of metastatic lesions. Furthermore, among patients with metastases detected by both methods, [^18^F]AlF-NOTA-FAPI-04 PET/CT revealed a greater number of metastatic lesions. These results highlight the significant advantage of this tracer combined with whole-body imaging for comprehensive systemic staging. Nevertheless, it must be cautiously noted that the CE-MRI in this study was confined to the abdominal region, and its inherent limited field of view intrinsically confers an advantage on whole-body PET/CT in detecting distant metastases. Consequently, the observed detection gain is primarily attributable to differences in imaging coverage, rather than solely to the superior diagnostic sensitivity of the FAPI tracer. This bias should be fully taken into account when interpreting the results.

Consistent with prior reports (17, 18), our results show that [^18^F]AlF-NOTA-FAPI-04 PET/CT achieves high sensitivity for detecting primary tumors, with a detection rate comparable to CE-MRI. This performance likely reflects the characteristic desmoplastic stroma and cancer-associated fibroblasts (CAFs) infiltration in biliary tract malignancies, where high FAP expression provides a specific target for FAPI imaging. We also assessed whether anatomic subsite and histologic differentiation influenced FAPI uptake and found no significant associations with tracer intensity. This contrasts with Guo et al. (18), who reported a positive correlation between FAPI uptake and histologic grade in hepatocellular carcinoma. This difference may be due to the inherent fibrotic tumor microenvironment of BTC, which may weaken the variation of FAPI uptake with grading. While higher tracer uptake enhances lesion conspicuity, it may also increase false-positive findings. Histopathology-proven cholangitis in two cases manifested as FAPI-positive and FDG-negative on PET/CT, likely due to inflammation-induced fibrosis (19). Conversely, two distal cholangiocarcinomas were false negatives. Because [^18^F]AlF-NOTA-FAPI-04 PET/CT is predominantly excreted via the biliary system, intense activity in the bile ducts and gallbladder can obscure lesions (12). In addition, tumor-associated pancreatitis or pancreatic fibrosis can produce diffuse pancreatic uptake (20, 21), markedly lowering the target-to-background ratio for distal cholangiocarcinoma in the pancreatic head. These factors underscore the limitations of this tracer for evaluating lesions in the peri-pancreatic biliary region.

MRI primarily evaluates lymph node metastases based on size and morphological characteristics. In this study, six false-positive cases of lymph node metastases in the abdominal region were detected by MRI. The reactive hyperplasia of lymph nodes reduces the specificity of metastasis assessment when relying on size-based criteria (14). In contrast, [^18^F]AlF-NOTA-FAPI-04 PET/CT, by specifically targeting tumor-associated fibroblasts, offers molecular-level functional imaging and demonstrates significant advantages in detecting metastatic lymph nodes across the body. Nevertheless, one lymph node metastasis confirmed by postoperative pathology was missed by both imaging modalities. It is important to note that FAPI PET imaging reflects FAP expression in the tumor microenvironment rather than directly characterizing tumor cells (9). In cases where metastases are driven mainly by tumor cell proliferation with minimal stromal remodeling and CAF activation, low FAP expression may lead to false-negative findings. Furthermore, small metastases may escape detection owing to their size and PET/CT spatial resolution and partial-volume limitations (6, 22).

The study findings indicate that [^18^F]AlF-NOTA-FAPI-04 PET/CT outperformed CE-MRI in detecting distant metastatic lesions, although no statistically significant difference in overall diagnostic performance was observed between the two imaging modalities, which may be attributed to the limited sample size. The advantage of PET/CT lies in its ability to identify additional metastatic lesions beyond the upper abdominal scanning range, which could have important clinical implications for M staging and treatment strategy decisions. It is noteworthy that two cases of false-positive bone uptake were identified by FAPI imaging in this study, which were later attributed to fractures and fibrous dysplasia based on follow-up. FAPI uptake in benign bone lesions is a common finding, frequently reported in conditions such as degenerative changes (20), fractures (23), and fibrous dysplasia (24). Additionally, one case in this cohort exhibited high FAPI uptake in a spinal meningeal lesion, while another showed mild-to-moderate FAPI uptake in multiple splenic lesions. Clinical follow-up and MRI findings supported the diagnoses of meningioma and hemangioma, respectively. Previous studies have confirmed that FAPI uptake can occur in both meningiomas (25) and splenic hemangiomas (26), likely due to the presence of activated fibroblasts within the tumor tissue. The interpretation of imaging results should be integrated with clinical information and follow-up to improve diagnostic accuracy.

Traditional [^18^F]FDG PET/CT has been widely utilized in oncology for whole-body imaging and metastatic lesion detection. However, its sensitivity in detecting extrahepatic cholangiocarcinoma remains relatively low (27), which limits its clinical application to some extent. In this study, [^18^F]AlF-NOTA-FAPI-04 PET/CT demonstrated high SUVmax and target-to-background ratio (TBR) in both primary and metastatic lesions, and it was significantly superior to [^18^F]FDG PET/CT in the paired subgroup analysis. This finding is consistent with previous studies (17).

Based on the findings of this study, whole-body PET/MRI demonstrates substantial potential in biliary tract malignancies. By integrating the molecular targeting capability of FAPI PET with the superior soft-tissue contrast and multiparametric imaging features of MRI, this approach holds promise for achieving precise staging of biliary tract cancers. MRI sequences such as contrast-enhanced imaging and DWI can help differentiate inflammatory uptake from tumor infiltration, thereby compensating for the relatively high false-positive rate associated with FAPI PET/CT alone. However, owing to inherent signal loss in the lung parenchyma and respiratory motion artifacts, the sensitivity of whole-body PET/MRI for detecting small pulmonary metastases is inferior to that of low-dose CT, which represents the major technical bottleneck for its clinical application.

This study has several limitations. Firstly, this study is a single-center retrospective study with a limited sample size (*n* = 46), which may reduce the generalizability and statistical power of the results. It should be noted that only a small subgroup of patients (*n* = 5) underwent paired [^18^F]FDG PET/CT, which precluded a comprehensive three-modality diagnostic comparison. Future studies with larger cohorts incorporating all three imaging modalities would be valuable to establish the optimal imaging strategy for BTC. Second, some lesions lacked pathological gold standards, relying instead on imaging and follow-up as surrogate references, which may introduce verification bias. Future prospective multicenter studies are warranted to confirm these findings and could integrate PET/MR technology to enhance soft tissue resolution.

## 5 Conclusion

In this retrospective study, we demonstrated that [^18^F]AlF-NOTA-FAPI-04 PET/CT and CE-MRI exhibit comparable sensitivity in detecting primary and metastatic lesions in biliary tract cancer. Notably, [^18^F]AlF-NOTA-FAPI-04 PET/CT showed superior specificity and diagnostic accuracy in lymph node detection and effectively addressed the limitations of abdominal CE-MRI in evaluating distant metastases across the entire body. These findings highlight the potential of [^18^F]AlF-NOTA-FAPI-04 PET/CT in enabling more precise staging and guiding personalized clinical decision-making.

## Data Availability

The original contributions presented in the study are included in the article/supplementary material, further inquiries can be directed to the corresponding authors.
